# Mind-Reading and Behavior-Reading against Agents with and without Anthropomorphic Features in a Competitive Situation

**DOI:** 10.3389/fpsyg.2017.01071

**Published:** 2017-07-07

**Authors:** Kazunori Terada, Seiji Yamada

**Affiliations:** ^1^Department of Electrical, Electronics and Computer Engineering, Faculty of Engineering, Gifu UniversityGifu, Japan; ^2^Digital Content and Media Sciences Research Division, National Institute of InformaticsTokyo, Japan; ^3^Department of Informatics, The Graduate University for Advanced Studies (SOKENDAI), HitotsubashiChiyoda, Tokyo, Japan

**Keywords:** mind-reading, behavior-reading, competitive game, behavioral variability, robotics, human robot interaction

## Abstract

Humans use two distinct cognitive strategies separately to understand and predict other humans' behavior. One is mind-reading, in which an internal state such as an intention or an emotional state is assumed to be a source of a variety of behaviors. The other is behavior-reading, in which an actor's behavior is modeled based on stimulus-response associations without assuming internal states behind the behavior. We hypothesize that anthropomorphic features are key for an observer switching between these two cognitive strategies in a competitive situation. We provide support for this hypothesis through two studies using four agents with different appearances. We show that only a human agent was thought to possess both the ability to generate a variety of behaviors and internal mental states, such as minds and emotions (Study 1). We also show that humans used mixed (opposing) strategies against a human agent and exploitative strategies against the agents with mechanical appearances when they played a repeated zero-sum game (Study 2). Our findings show that humans understand that human behavior is varied; that humans have internal states, such as minds and emotions; that the behavior of machines is governed by a limited number of fixed rules; and that machines do not possess internal mental states. Our findings also suggest that the function of mind-reading is to trigger a strategy for use against agents with variable behavior and that humans exploit others who lack behavioral variability based on behavior-reading in a competitive situation.

## 1. Introduction

Humans sometimes attribute minds to intelligent machines, as shown by the case of HAL in the film “2001: A Space Odyssey.” To what types of agent do humans attribute minds? According to the Machiavellian intelligence hypothesis, human intelligence has been evolutionarily shaped, and the mind developed to handle complex social environments (Byrne and Whiten, 1988). Attributing mind to others is necessary to cope with both cooperators and competitors. In the present study, we address mind attribution to intelligent agents in a competitive situation.

One of the main properties of intelligence is the ability to generate unlimited behavioral patterns to reach a given goal (Byrne, 1995; Roth and Dicke, 2005). This ability, which is also known as searching or exploration (Newell et al., 1959; Sutton and Barto, 1998), enables agents to find novel ways to adapt to a given environment. The optimal way to cope with this type of intelligent agent, which has behavioral variability in both competitive and cooperative situations, is to attribute abstract mental states to it as the causes of its behavior, as in mind-reading (Whiten, 1996), a theory of mind (Premack and Woodruff, 1978), or an intentional stance (Dennett, 1987). The advantages of attributing abstract causal mental states are not only the reduction in the cognitive complexity required to understand another's behavior but also the prediction of the other's future behavior (Heider, 1958; Dennett, 1987; Gergely et al., 1995; Whiten, 1996; Gergely and Csibra, 2003; Call and Tomasello, 2008).

**Figure 1:**
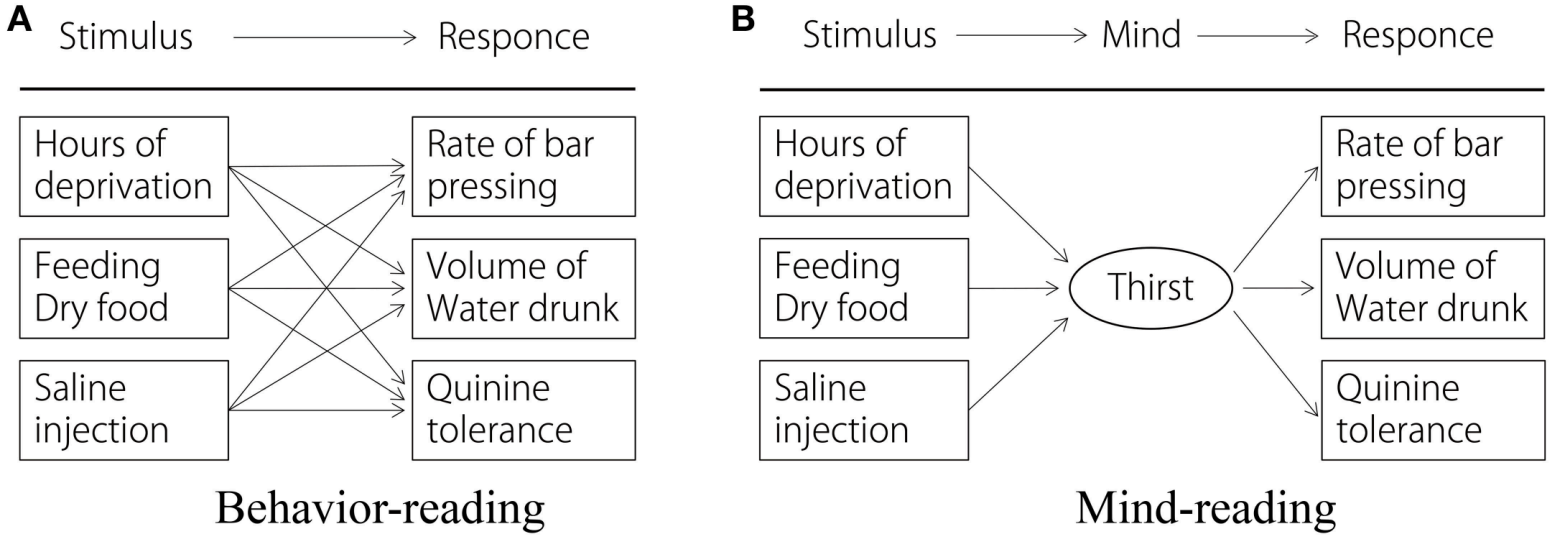
Comparison of behavior-reading **(A)** and mind-reading **(B)** (modified and redrawn from Whiten, 1996). An internal state (mind) is used to explain the other's behavior as a source of variety of behavior and to predict future behavior in a new situation.

An alternative strategy to mind-reading is behavior-reading, in which an actor's superficial behavior is modeled based on a finite set of state-action (stimulus-response) associations (Krebs and Dawkins, 1984; Dennett, 1987; Whiten, 1996; Call, 2003; Povinelli and Vonk, 2003; Call and Tomasello, 2008). The crucial difference between these two strategies is whether or not an abstract internal state such as an intention or an emotional state is assumed (see Figure 1). An abstract internal state is used to represent multiple state-action associations. Predicting another's actions using behavior-reading is difficult, especially when the observed situation is novel, i.e., the state-action rule is not recorded in the database. However, the mind-reading strategy is effective for predicting another's future behavior in a new situation. Once the actor's intention and current situation have been captured, the mind-reading strategy enables one to infer the actor's future behavior by applying the rationality principle, which is the common assumption that an actor utilizes the most effective way to reach a goal (Gergely et al., 2002; Gergely and Csibra, 2003). For example, if an observer detects an actor's intention as being “quench his/her thirst,” the observer predicts that he/she will produce a suitable action such as inserting coins into a vending machine, opening a faucet, boiling seawater to obtain fresh water, etc., which is rational given the current constraint. This prediction is difficult unless the internal state is not assumed.

Behavior-reading is effective in a competitive situation when the opponent cannot generate unlimited behavioral patterns. One can develop theories by observing statistical behavioral patterns when the opponent's behavior is composed of a finite set of behavioral rules (Krebs and Dawkins, 1984). Then, he/she can generate a counter-plan to exploit the opponent's behavior. However, behavior-reading does not work well when both competitors have the capability to generate unlimited behavioral patterns. If one identifies a rule that governs the competitor's behavior and applies an exploitative strategy to the competitor's behavioral rule, the strategy is countered by a more sophisticated strategy. Any strategy that can be modeled based on regularity or statistical patterns, i.e., simple state-action associations, such as making the same choice in every game of rock-paper-scissors, could be exploited by opponents who are capable of sophisticated behavior-reading. Deception is a specific act that utilizes behavior-reading. In the deceiving act, the actor makes others believe false information to exploit the others' state-action associations. That is, if a victim has a tendency to produce action *A* when it receives information *I*, a deceiver can profit from *A* by providing *I*, even if *I* is not true. Once the victim realizes that the false information is produced algorithmically, he/she can adopt a counter-algorithmic strategy to avoid being exploited. However, if the deceiver is able to generate further strategic changes, it is dangerous for the victim to adopt a specific algorithmic strategy.

The only way to avoid prediction by behavior-readers is ensure that one's own behavior is random (a “protean strategy”) (von Neumann and Morgen, 1944; Nash, 1950; Humphries and Driver, 1970; Miller, 1997). In the case of the game of rock-paper-scissors, it is known that there is a unique mixed Nash equilibrium in which each player selects the three available actions with equal probability (van den Nouweland, 2007). In the case of opposing deceivers, an effective strategy for the victim is to generate completely unpredictable behavior, i.e., random action, to avoid the behavior being modeled by the deceivers.

Assuming that a mind is a source of variety in behavior is important in a competitive situation because one then stops using behavior-reading and generates an opposing strategy. Therefore, mind attribution may act as a cue for triggering a mixed strategy. Neuroimaging studies have suggested that the brain regions relating to the theory of mind are activated when humans must predict and understand one another's behavior in competitive situations (Gallagher et al., 2002; Decety et al., 2004). Furthermore, Takahashi et al. (2014) and Takahashi et al. (2015) have reported that the brain regions relating to the theory of mind are activated and that participants generate more random responses when they believe that they are playing a competitive game with a human rather than a computer.

Researchers have argued that behavioral variability might be a cue for mind attribution. In studies by Luo and Baillargeon (2005) and Shimizu and Johnson (2004), infants attributed intentions to an agent when he/she chose his/her actions freely. In Bíró and Leslie (2007), the most effective indication of goal directedness was the variability of the strategy used to achieve the goal. According to Csibra and Gergely (2007), “Evidence for ‘freedom’ and for the capability of changing the course of action seems to be sufficient for infants to identify an object as an agent and to treat it as worthy of goal attribution.” An agent with an appearance that recalls behavioral variability might be a direct cue for intention attribution. Furthermore, the unpredictability that is used to represent the subjective evaluation of a variety of behavior is thought to be a cue for triggering anthropomorphism (Epley et al., 2007; Waytz et al., 2010).

From the above discussion, we hypothesized that the presence of an opponent's ability to generate unlimited behavioral patterns in competitive situations leads to different cognitive and behavioral strategies. More precisely, humans attribute minds to agents with behavioral variability and superficial rules to agents without behavioral variability. The difference in the cognitive strategies leads humans to use different behavioral strategies. Mind attribution contributes to the production of mixed strategies, and rule attribution contributes to the production of exploitative strategies in competitive situations.

To test the hypothesis, we conducted two studies. In Study 1, we investigated whether the appearances of the agents contributed to humans' recognition of anthropomorphic features such as possessing behavioral variability, mind, intelligence, and complexity. The main purpose of Study 1 is to preliminarily investigate what types of anthropomorphic features people recognize from the appearances of the agents used in Study 2. In Study 2, we tested our main hypothesis. We investigated whether mind-reading and behavior-reading result in different decisions being made in a zero-sum game in which players are allowed to use both exploitative and mixed strategies.

## 2. Ethics statement

Studies 1 and 2 were both carried out in accordance with the recommendations of the Ethical Guidelines for Medical and Health Research Involving Human Subjects provided by the Ministry of Education, Culture, Sports, Science and Technology and Ministry of Health, Labor and Welfare in Japan with written informed consent from all participants. All participants gave written informed consent in accordance with the Declaration of Helsinki. The protocol was approved by the Medical Review Board of Gifu University Graduate School of Medicine.

## 3. Study 1

In Study 1, we asked participants to rate agents in a video on scales of *behavioral variability* and *intelligence*. The aim of this study was to investigate the connection between an agent's appearance and the subjective impression of its behavioral variability. Furthermore, the connection of appearance with intelligence and mind was assessed.

### 3.1. Methods

#### 3.1.1. Participants

Twenty-five graduate and undergraduate students attending Gifu University in Japan (17 male, 8 female, *M*_*age*_ = 21.9 years, *SD*_*age*_ = 1.5 years, age range: 19–24 years) participated in the study.

#### 3.1.2. Materials and procedures

**Figure 2:**
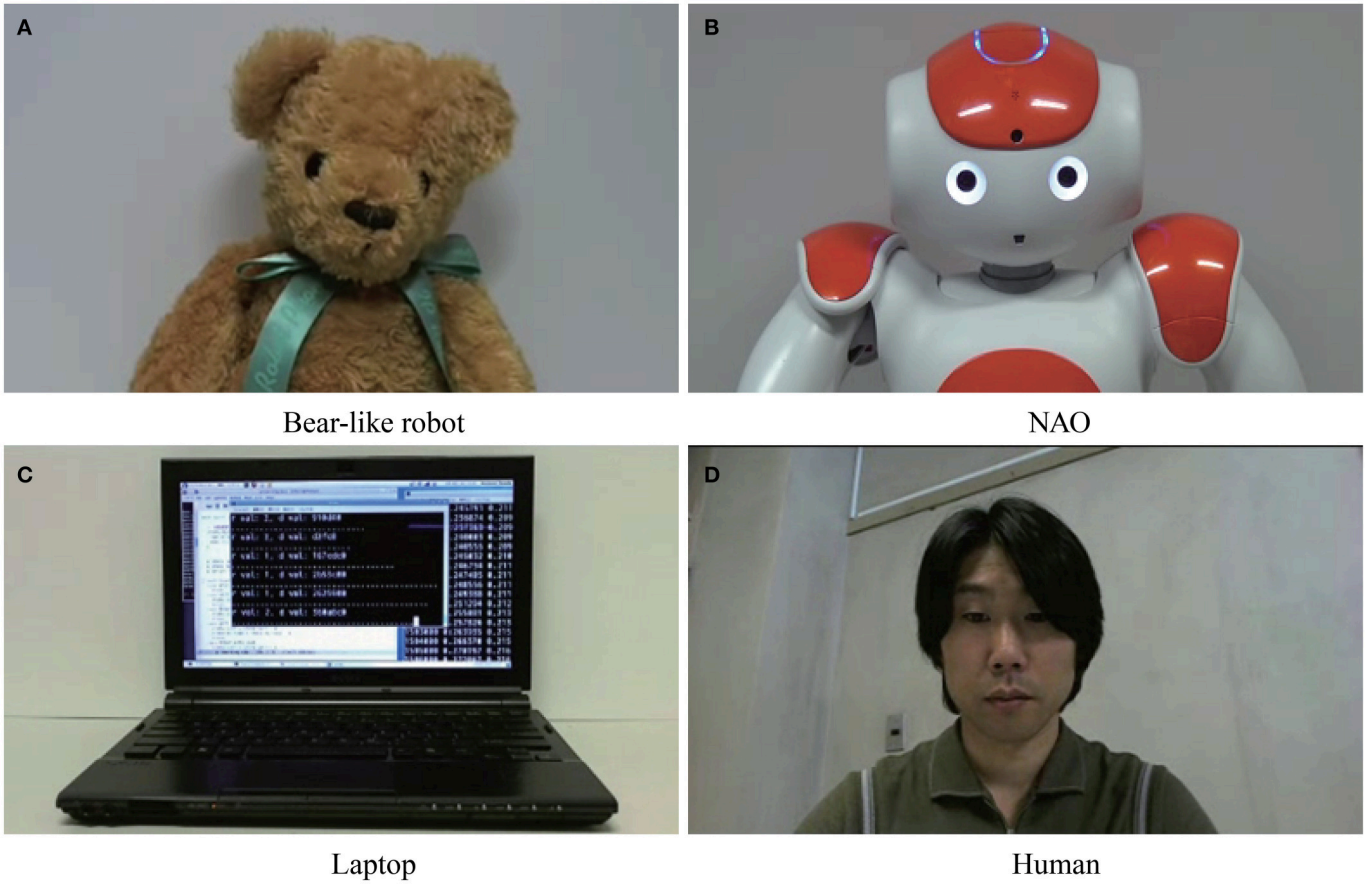
The four agents used as opponents in the game. Informed consent to publish the portrait was obtained from the person in **(D)**. **(A)** Bear-like robot. **(B)** NAO. **(C)** Laptop. **(D)** Human.

The participants completed an on-line survey that asked them to rate the behavior of four agents using a 20-item questionnaire. Each item was rated on a seven-point Likert scale from 1 to 7. The four agents were the laptop, the bear-like robot, NAO (Aldebaran Robotics), and the human agent shown in Figure 2. The movie was located at the top center of the web browser. The size of the movie was 640 px × 480 px. At the bottom of the movie, the following instructions were shown: “Please answer the following questions based on the assumption that you are playing a competitive game with the agent shown in the video.” The questionnaire was located below the movie on the same page. In the video, the bear-like robot and NAO moved their heads as if they were playing an on-line game. The human's behavior was recorded in advance as he played the game used in Study 2. The laptop output lines of text as if were calculating something strategic. The 20 items were *is complex, is mechanical, is predictable, is unpredictable, has a limited behavioral pattern, acts according to predefined rules, acts according to a small number of rules, is intelligent, is resourceful, is witty, is insightful, is knowledgeable, thinks logically, is able to concentrate, is able to make decisions, has a mind, is able to read one's mind, is goal-directed, and is emotional*. These items were selected to clarify the difference between humanness and machineness in a human's concept. We specifically included items regarding behavioral variability, intelligence, mind, complexity and predictability.

### 3.2. Results

We ran a factor analysis with promax rotation on the participants' ratings of the 20 items to determine the underlying factorial structure. The analysis produced a three-factor solution (with eigenvalues >1.0) that explained 71% of the variance and included three factors (see Table 1). Factor 1, which was labeled “personal intelligence,” included items such as *is able to concentrate, thinks logically, is knowledgeable, is able to make decisions, is witty, is intelligent, is able to anticipate the future, is goal-directed*, and *is resourceful*. Factor 2, which was labeled “social intelligence,” included items such as *has a mind, is emotional, is mechanical* (negative), *has a limited behavioral pattern* (negative), *acts according to pre-defined rules* (negative), *is insightful*, and *is able to read one's mind*, Factor 3, which was labeled “unpredictability,” included items such as *is predictable* (negative), *acts according to a small number of rules* (negative), *is unpredictable*, and *is complex*.

**Table 1:** Factor loadings and factor correlations.

**Item**	**Factor 1 (personal intelligence)**	**Factor 2 (social intelligence)**	**Factor 3 (unpredictability)**
Is able to concentrate	0.90		
Thinks logically	0.87		
Is knowledgeable	0.82		
Is able to make decisions	0.75		
Is witty	0.70		
Is intelligent	0.66		
Is goal-directed	0.44		
Is resourceful	0.41		
Is insightful	0.47	0.52	
Has a mind		1.01	
Is emotional		0.95	
Is mechanical		−0.79	
Has a limited behavioral pattern		−0.54	−0.42
Acts according to pre-defined rules		−0.52	−0.41
Is predictable			−0.83
Acts according to a small number of rules			−0.63
Is unpredictable			0.58
Is able to read one's mind			0.42
Eigenvalue	9.78	2.43	1.19
Percent of variance explained	51.50	12.79	6.27
Factor 2	0.49		
Factor 3	0.50	0.65	
Factor loadings <0.40 were suppressed.			

**Figure 3:**
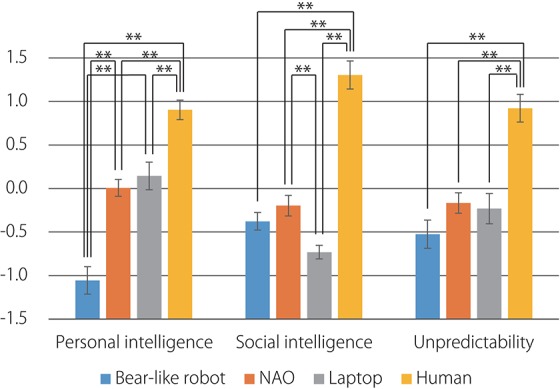
Comparison of the mean factor scores for the four agents. Error bars represent standard errors. ^*^p < 0.05; ^**^p < 0.01.

The comparison of the mean factor scores for the four agents is presented in Figure 3. The results of a one-way analysis of variance (ANOVA) revealed a significant difference in the mean factor scores of the four agents on the “personal intelligence” factor [*F*_(3, 72)_ = 38.78, *p* < 0.001], the “social intelligence” factor [*F*_(3, 72)_ = 56.38, *p* < 0.001], and the “unpredictability” factor [*F*_(3, 72)_ = 20.80, *p* < 0.001]. *Post-hoc* tests on the “personal intelligence” factor using the Bonferroni correction revealed that this factor's mean score for the human agent was significantly higher than those for the bear-like robot, NAO, and laptop and that the mean factor score for the NAO and laptop were significantly higher than those for the bear-like robot. Similarly, for the “social intelligence” factor, the mean factor score for the human agent was significantly higher than those for NAO, the bear-like robot, and the laptop, and that for the NAO was significantly higher than that for the laptop. Furthermore, for the “unpredictability” factor, the mean factor score for the human agent was significantly higher than those for the bear-like robot, NAO, and the laptop.

#### 3.2.1. Discussion

The factor analysis revealed that behavioral variability, which we assume is one of the main properties of intelligence, was excluded from the “personal intelligence” factor that contained other properties, such as logical thought, decision making, and knowledge. This might be because although behavioral variability is an important factor in producing an alternative way to reach goals and is explicitly modeled in artificial intelligence algorithms (Newell et al., 1959; Russell and Norvig, 1995; Sutton and Barto, 1998), it is not included in the folk concept of “intelligence.” Instead, items that indicate behavioral variability such as *is not mechanical, does not have a limited behavioral pattern*, and *does not act according to predefined rules* were included in the “social intelligence” factor. This finding indicates that the ability to generate a variety of behaviors and the presence of internal states, such as a mind and emotions, are related in the folk concept. This is consistent with the findings of studies in which infants attributed intentions to agents with behavioral variability (Shimizu and Johnson, 2004; Luo and Baillargeon, 2005; Bíró and Leslie, 2007; Csibra and Gergely, 2007).

Although the “unpredictability” factor was extracted as an independent factor, it was correlated with the “social intelligence” factor (the factor correlation was 0.65). This correlation is understandable because the “social intelligence” factor included items, such as *does not have a limited behavioral pattern* and *does not act according to predefined rules*, that are objective properties of the entities that induce a subjective impression of “unpredictability.”

Analyzing the factor scores revealed that the human agent was thought to possess higher personal intelligence, social intelligence, and unpredictability than the three artifacts. It is notable that the bear-like robot was rated lower than NAO and the laptop in terms of the “personal intelligence,” whereas it was rated as the same as NAO and the laptop in terms of the “social intelligence” and “unpredictability,” indicating that the factors that differentiate the human agent from the artifacts were “social intelligence” and “unpredictability.”

In summary, our findings show that humans understand that human behavior is varied; that humans have internal states, such as minds and emotions; that the behavior of machines is governed by a limited number of fixed rules; and that machines do not possess internal mental states.

## 4. Study 2

This study tested whether the appearances of different agents contributed to humans' use of mixed or exploitative strategies in a competitive situation. In the experiment in Study 2, the opposing agents produced an easily predictable sequence of actions to make the participants behave in fixed patterns. Then, the opponents suddenly changed their action sequences to exploit the participants' fixed behaviors. The best response to this change in action was to quickly adapt to the new action sequence because the opponent had never changed the action sequence. However, the participants did not know this fact. Therefore, we made the following predictions: If participants understand that the opponent's change in action is caused by a mind, they will be cautious because they will expect further change and will not easily adapt to a new action sequence, i.e., they will follow a mixed strategy. If the deceptive behavior is believed to be algorithmic, a human will expect to observe a fixed pattern in subsequent behaviors.

### 4.1. Method

#### 4.1.1. Participants

Eighty-five graduate and undergraduate students attending Gifu University in Japan (48 male, 37 female, *M*_*age*_ = 21.56 years, *SD*_*age*_ = 1.45 years, age range: 20–25 years) participated in the study. The participants were randomly assigned to four opponents. The participants were unaware of the actual purpose of the study. Instead, they were informed that the aim of the study was to assess the usability of an on-line game system. The participants were also told that they would win a book coupon whose value was based on their score. A single-factor, four-level, between-subjects experimental design was used.

##### 4.1.1.1. Materials and procedure

A repeated penny-matching game with bonus rounds was used to test the hypothesis. The ordinary penny-matching game is a zero-sum game for two players, A and B. Each player has a penny and must secretly turn the penny over to show heads or tails. The players then reveal their choices simultaneously. If the pennies match (both heads or both tails), player A keeps his or her penny and is allowed keep player B's penny (+1 for A, −1 for B). If the pennies do not match (one heads and one tails), player B keeps both pennies (−1 for A, +1 for B). This game has no pure-strategy Nash equilibrium. However, the game does have a unique mixed-strategy Nash equilibrium because each player chooses heads or tails with equal probability.

We modified the rules of the game to allow the players to use deceptive strategies. Each game consisted of six rounds. The payoff in the sixth round (the bonus round) was increased 20-fold. The unique Nash equilibrium of the penny-matching game was mixed (random), even with the added bonus round. The agents acting as opponents in our study used only two strategies: straightforwardness and deception. The algorithms underlying the straightforward and deceptive strategies are as follows:

- *A1: (Straightforward)* Select a side regularly during the first five rounds and follow this regularity when selecting a side during the sixth round.
- *A2: (Deceptive)* Select a side regularly during the first five rounds and *do not* follow this regularity when selecting a side during the sixth round.

Each participant played 15 games. The straightforward strategy was used in the first three games, and the deceptive strategy was used in the remaining 12 games. Four series of choices were used for the straightforward strategy. They were implemented with a uniform pattern in which the same side was always selected or with an alternating pattern in which heads and tails were selected in turn: HHHHHH, TTTTTT, HTHTHT, THTHTH. Four series of choices were used for the deceptive strategy. They were implemented by simply violating the regularity of the straightforward strategy in the sixth round: HHHHHT, TTTTTH, HTHTHH, THTHTT. A series of choices was randomly selected for each game.

The series of choices with fixed patterns in this game were the same as those in normal zero-sum games and were easily exploited by players who were able to read behavior. In this study, the opposing agents used the straightforward strategy in the first three games. The straightforward strategy was simple to exploit because it consisted of a series of choices with simple regularity. However, in the fourth game, the opposing agents suddenly switched from the straightforward strategy to the deceptive strategy. As a result, those who exploited the opponent during the first three games would lose the sixth round of the fourth game. Although the algorithmic difference between the two strategies was slight, changing strategies would give a strong impression of having mind. The simple regular pattern in the first five rounds would be then be construed as a trapping pattern that misled the player into making the wrong prediction in the sixth round because of the large change in the payoff. Therefore, if the participants attributed a mind to the strategy change in the fourth game, they were expected to use a mixed strategy in the remaining games. In contrast, if the participants construed the strategy change as merely a change in the rules, they were expected to exploit the deceptive strategy.

**Figure 4:**
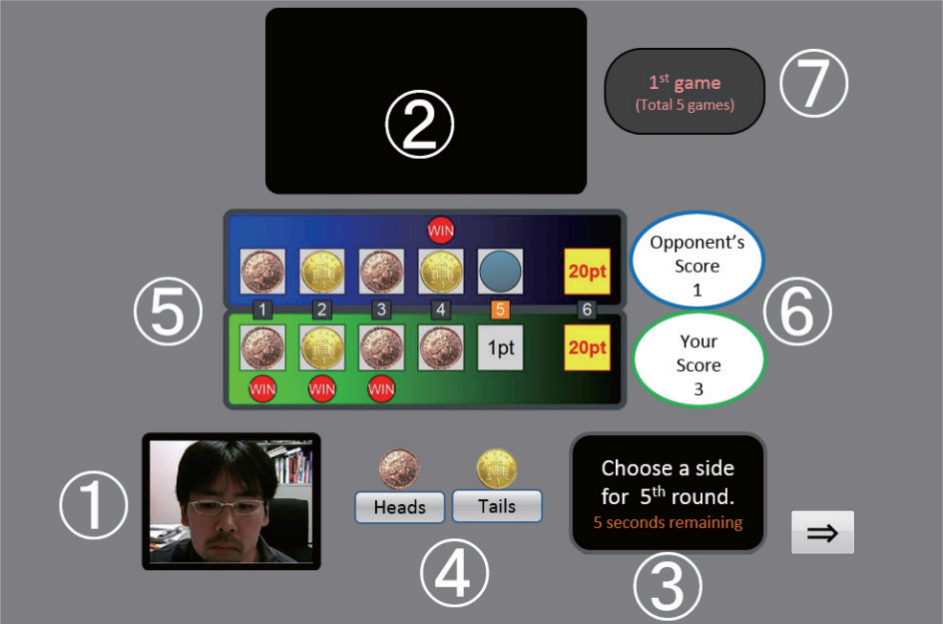
Interface of the on-line experimental system: (1) the participant's face, (2) a video of the opponent agent, (3) the time remaining, (4) the choice buttons, (5) both players' previous choices, (6) the players' scores, and (7) the game number. We used a Japanese version in our study.

The game was designed using JavaScript and HTML and was played in a web browser (Firefox). Figure 4 shows the game interface. A Flash video of the opponent (the same four types of opponents were used as in Study 1) was displayed at the top of the interface. The agents' behaviors, such as choosing which side of the coin to show, were automatically controlled by the JavaScript program. The participants were told that the opponent was on-line. To make the participants believe that they were playing an on-line game, not only the opponent's appearances but also the participant's face, captured by a web camera mounted on the monitor, weredisplayed on the interface. The participant's face was shown at the bottom left of the interface. The participants were instructed to click the button that corresponded to their choice within 10 s in each round. Both players' scores were displayed on the interface, and both players' choices remained displayed so that the participants could identify their opponents' strategies.

The participants were seated in front of desktop computers. They were asked to read the instructions on the web page and to play the game. Before beginning the experiment, each participant completed five training games.

##### 4.1.1.2. Measurement

The outcome of the sixth round in each of the 15 games was recorded because each participant's adaptation to the opponent's strategy provided the salient data in this study.

### 4.2. Results

**Figure 5:**
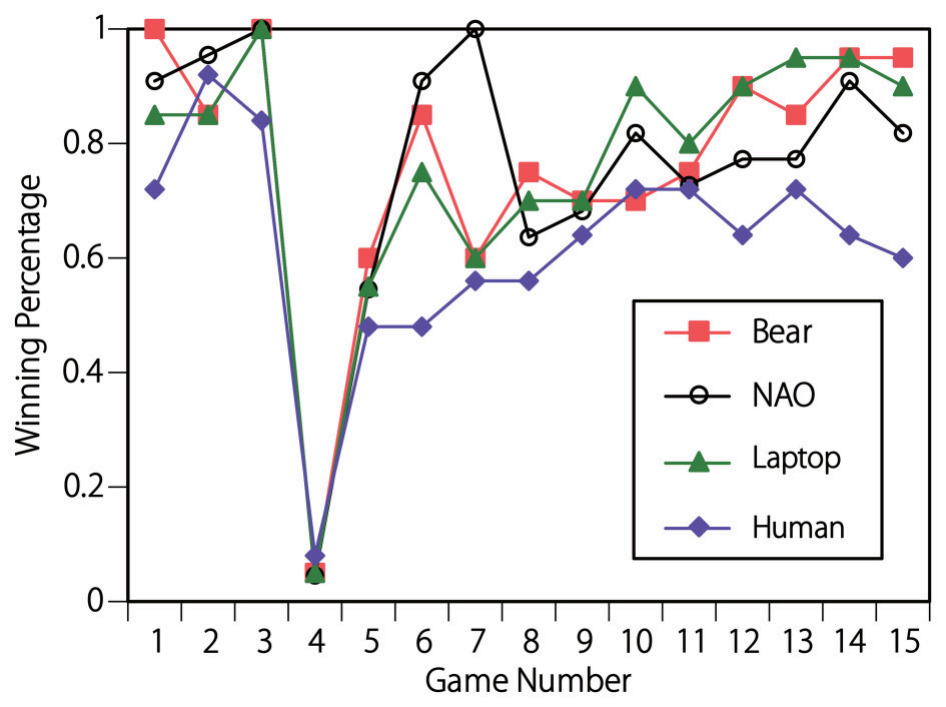
Percentage of participants who won the sixth round in each of the 15 games. After the fifth game, the mean percentage of sixth rounds won in the human agent condition was significantly lower than that in the other conditions, which indicates that those who played against the human agent used a mixed strategy against their opponent, whereas those who played against NAO, the laptop, and the bear-like robot used an exploitative strategy against their opponent.

**Figure 6:**
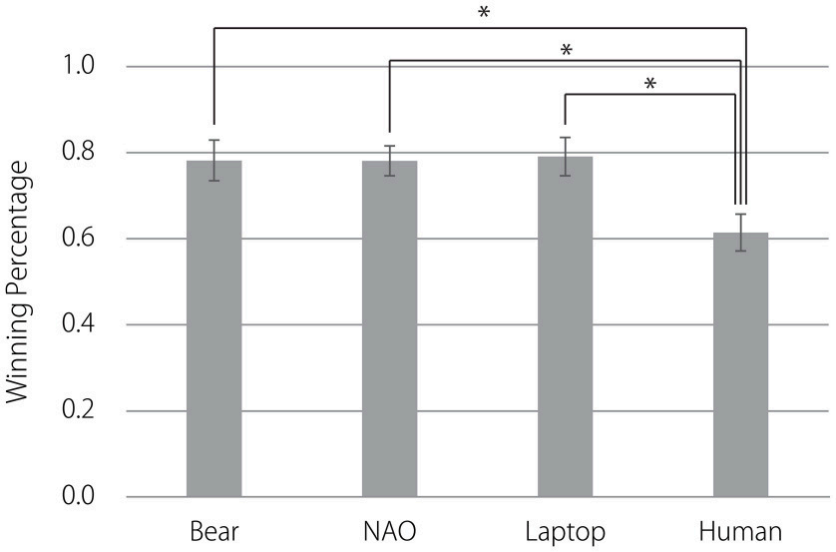
Comparison of the mean percentage of sixth rounds won in the fifth through fifteenth games for the four opponents. Error bars represent standard errors. ^*^p < 0.05.

The percentage of participants who won the sixth roundin each game was plotted against the game number and is shown in Figure 5. In the first three games, the subjects' behavior appears to be identical across the four conditions. This is confirmed statistically. A one-way ANOVA [*F*_(3, 83)_ = 1.96, *p* = 0.13] confirmed that there was no significant difference in the mean percentage of sixth rounds won during the first three games under each of the four conditions.Nearly all of the participants lost the sixth round of the fourth game because in that game, the agents changed their strategies from straightforward to deceptive. After the fourth game, the winning percentage in all conditions recovered to a level above 50%. However, the behavior of subjects in the human agent condition differed from that of the other three conditions. A one-way ANOVA [*F*_(3, 83)_ = 4.20, *p* < 0.01] confirmed a significant difference in the mean percentage of sixth rounds won in the fifth through fifteenth games under each of the four conditions (see Figure 6). A Fisher's LSD *post-hoc* test revealed that the mean winning percentage was significantly lower when the opponent was the human agent than it was when the opponent was the bear-like robot, NAO, or the laptop.

### 4.3. Discussion

Study 2 was conducted to test whether the appearances of different agents contributed to humans' use of mixed or exploitative strategies in a competitive situation. This is explained directly by the winning percentage for the sixth round after fourth game. Winning the sixth round indicated that the participants' expectations regarding their opponents' strategy were correct and that they exploited their opponents. Losing the sixth round indicated that the participants did not exploit their opponents' behavior and instead used a mixed strategy. The mean percentage of sixth rounds won from the fifth to the fifteenth game played against the human agent was significantly lower than those of games played against the bear-like robot, NAO, and the laptop. Thus, the participants who played against the bear-like robot, NAO, and the laptop exploited the opponent's deceptive strategy, whereas those who played against the human agent did not exploit their strategy but instead used a mixed strategy.

However, the mean percentages of sixth rounds won during the first three games did not differ across the four conditions, indicating that participants in all four conditions exploited their opponents' straightforward strategies until the opponent produced the deceptive strategy. This is inconsistent with the facts reported in previous studies. In competitive situations, humans are known to generate more random responses when they believe that they are playing a game with a human agent rather than a computer (Takahashi et al., 2014, 2015). However, all of the participants who participated in all of the conditions used exploitative (non-random) strategies in the first three games, and only those who participated in the condition involving a human agent tended to produce a mixed strategy after the opponent's deceptive act. The inconsistency between our results and those of Takahashi et al. might be attributable to differences in the opponent's strategy. In Takahashi et al. (2014, 2015), the opposing agents chose randomly, and thus, the participant did not have the option of exploiting the opponent's behavior. Our results and those of Takahashi et al. suggest that a combination of categorical knowledge and the opponent's exploitative behavior is a cue for using a mixed strategy when the opponent's strategy is understood algorithmically.

## 5. General discussion

The hypothesis of our study was that anthropomorphic features are key for an observer switching between mind-reading and behavior-reading in a competitive situation when he/she understands and predicts an opponent's behavior. Specifically, we focused on the ability to generate unlimited behavioral patterns and having minds as the anthropomorphic features. We predicted that humans would use mind-reading against agents with behavioral variability and behavior-reading against agents without behavioral variability. Behavioral variability was recognized by the participants visually when they were shown agents of different types. The results of Study 1 confirmed that the recognition of behavioral variability depended on the agent's appearance. Participants rated the behavioral variability of the human agent higher than those of the other agents (a bear-like robot, NAO, and a laptop). The results of Study 1 also revealed that in the concept of humanness, the assumption of behavioral variability is closely related to an assumption of an internal state. This finding indicates that humans tend to assume that although other humans possess internal states, such as minds, emotions and behavioral variability, machines neither possess internal states nor vary their behavior.

The results of Study 2 showed that humans tended to use mixed strategies against the agent that appeared human when it behaved deceptively in a competitive game but exploitative strategies against the agents that appeared as a bear-like robot, NAO, and a laptop. Taken together, the results of both studies confirm our prediction by implying that humans attribute minds to agents that are capable of generating a variety of behaviors and use mixed strategies against them when they produce deceptive behavior in a competitive situation. In contrast, humans attribute rules to agents without behavioral variability and use exploitative strategies.

Our results indicate that humans understand that the behavior of machines is based on a limited number of fixed rules and know that humans vary their behavior to reach goals. This is consistent with the results of previous studies (Meltzoff, 1995; Levin et al., 2013). Levin et al. (2013) conducted an experiment with a modified version of the location/object scenario described in Woodward (1998) and showed that adults made more predictions that intentions were underlying human behavior than the behavior of robots or computers. In Levin's experiment, an entity reached for one of two objects on a grid. The objects' locations were then swapped, and the participant was asked whether the entity would reach toward the old location or the new one. The adults predicted that humans would reach toward the new locations of the objects reached for in the last two trials but that robots and computers would reach toward the old locations, which then contained new objects that had not been reached for in the previous trials. These results indicated that adults assume that the reaching behaviors of computers and robots are defined by predefined rules, whereas humans are capable of generating alternative ways of reaching a goal.

The results of Meltzoff (1995), which were obtained through the failed attempt paradigm, are also similar to those of our experimental paradigm in terms of behavioral variability. He showed that 18-month-old children did not attribute intentions to the movements of a mechanical device by demonstrating that although the children were able to extrapolate and predict the sequence of behaviors involved in failed attempts made by a human, they were not able to do so when the attempts were made by a mechanical device. Thus, the children in the “mechanical device” experiment would have identified algorithms for a sequence of behaviors based on categorical knowledge of machines. As a result, they may have been unable to use alternative methods of extrapolating the behavior of the mechanical device.

Studies in developmental psychology suggest that behavioral variability might be a cue for attributing intentions to non-human agents (Shimizu and Johnson, 2004; Luo and Baillargeon, 2005; Bíró and Leslie, 2007; Csibra and Gergely, 2007). One possible explanation for this activity is motivation to reduce uncertainty. Epley et al. (2007) suggested that anthropomorphism, such as attributing intentions, goals, and emotional states to a non-human agent, is used to reduce uncertainty driven by the motivation to explain and understand the behavior of other agents to interact effectively and operate them (effectance motivation). Waytz et al. (2010) demonstrated that increasing effectance motivation by manipulating the perceived unpredictability of a non-human agent increases anthropomorphism. However, behavior-reading in which another's behavior is modeled based on stimulus-response (S-R) association may contribute further to reducing uncertainty. The modeling strategy might be selected according to the situation or the task. Our results indicate that in a competitive situation, humans first use behavior-reading offensively and then use mind-reading defensively when they realize that an exploitative strategy is no longer effective. In our study, behavioral variability was not directly manipulated as an independent variable. A study using agents with the same appearance and variable behavioral variability would reveal whether humans increasingly use mind-reading as behavioral variability increases.

We assumed that one of the main properties of intelligence is the ability to generate unlimited behavioral patterns to reach a given goal, and behavioral variability might be a cue for attributing intentions. However, this does not mean that anthropomorphism is reduced to behavioral variability. Whereas machines can generate random numbers, this is difficult for humans to do (Rapoport and Budescu, 1992, 1997), suggesting that machines can produce more variability than humans. Another important factor that differentiates humans from machines and is not explicitly considered in this study is the ability to evaluate whether an exploratory (random) action serves the actor's goal, i.e., rationality. Rationality has been reported as one of the cues that invoke intention attribution (Gergely et al., 1995; Kamewari et al., 2005; Csibra, 2008). Deception is a rational act that utilizes another's stimulus-response associations. It is possible that participants in the human agent condition perceived rationality in the human face and adopted a defensive strategy against the opponent.

The present study suffered from certain limitations. First, while our participants were selected from a small, culturally homogeneous population, and we did not control for the gender ratio, studies have suggested that gender (Baron-Cohen, 2010), age (Vetter et al., 2014), and culture (Frank and Temple, 2009) influence the ability to infer others' mental states. Larger and more diverse samples should be used to examine gender, age, and cultural effects on switching between mind-reading and behavior-reading. Second, we used the bear-like robot, NAO, and laptop as artifacts. Takahashi et al. (2014) suggest that a robot's appearance affects humans' strategies in a competitive situation and mind attribution. Specifically, participants in their experiment who were confronted with a robot with a sophisticated appearance, such as an android, responded in the same way as when confronted with a human. Further investigation in which the sophistication level is varied as an experimental factor should be performed to generalize these findings.

Our results suggest that within the confines of a competitive situation, mind-reading contributes to triggering an opposing strategy, indicating that participants predicted the opponent's future exploitative action by attributing an exploitative mind to it. This function of mind-reading can be generalized to a non-zero-sum situation in which cooperators and competitors (free riders) are mixed. In such a context, one should rapidly differentiate competitors from cooperators and avoid useless battles against competitors. Mind-reading is useful for this purpose. Mind attribution is used to evaluate agents as current or future allies or enemies by attributing harmful (exploitative) intentions or helpful (cooperative) intentions (Young and Waytz, 2013). Therefore, attributing abstract mental states to agents in a non-zero-sum situation might also be important. Further investigation using a non-zero-sum game should be performed to investigate whether anthropomorphic features contribute to an observer's decision to switch between mind-reading and behavior-reading in a non-zero-sum situation.

## Author contributions

KT and SY designed the experiments, analyzed the data and wrote the manuscript. KT performed the experiments.

### Conflict of interest statement

The authors declare that the research was conducted in the absence of any commercial or financial relationships that could be construed as a potential conflict of interest.
